# Microbial dysbiosis in oral squamous cell carcinoma: A systematic review and meta-analysis

**DOI:** 10.1016/j.heliyon.2023.e13198

**Published:** 2023-01-24

**Authors:** Xiaoyun Yu, Yongmei Shi, Rongtao Yuan, Zhenggang Chen, Quanjiang Dong, Linzi Han, Lili Wang, Jianhua Zhou

**Affiliations:** a Graduate School of Dalian Medical University, Dalian, 116044, China; b Department of Stomatology, Qingdao Municipal Hospital, Qingdao University, Qingdao, 266071, China; c Department of Outpatient, Qingdao Municipal Hospital, Qingdao University, Qingdao, 266071, China; d Central Laboratories and Department of Gastroenterology, Qingdao Municipal Hospital, Qingdao University, Qingdao, 266071, China

**Keywords:** Oral squamous cell carcinoma, Microbiome, *Fusobacterium*, *Streptococcus*, Systematic review, Meta-analysis

## Abstract

**Objective:**

The aim of this study was to summarize previously published data and assess the alterations in the composition of the oral microbiome in OSCC using a systematic review and meta-analysis.

**Design:**

Electronic databases were systematically searched for studies on the oral microbiome in OSCC published before December 2021. Qualitative assessments of compositional variations at the phylum level were performed. The meta-analysis on abundance changes of bacteria genera was performed via a random-effects model.

**Results:**

A total of 18 studies involving 1056 participants were included. They consisted of two categories of studies: 1) case-control studies (n = 9); 2) nine studies that compared the oral microbiome between cancerous tissues and paired paracancerous tissues. At the phylum level, enrichment of Fusobacteria but depletion in Actinobacteria and Firmicutes in the oral microbiome was demonstrated in both categories of studies. At the genus level, *Fusobacterium* showed an increased abundance in OSCC patients (SMD = 0.65, 95% CI: 0.43–0.87, Z = 5.809, *P* = 0.000) and in cancerous tissues (SMD = 0.54, 95% CI: 0.36–0.72, Z = 5.785, *P* = 0.000). The abundance of *Streptococcus* was decreased in OSCC (SMD = −0.46, 95% CI: −0.88−0.04, Z = −2.146, *P* = 0.032) and in cancerous tissues (SMD = −0.45, 95% CI: −0.78–0.13, Z = −2.726, *P* = 0.006).

**Conclusions:**

Disturbances in the interactions between enriched *Fusobacterium* and depleted *Streptococcus* may participate in or prompt the occurrence and development of OSCC and could be potential biomarkers for detection of OSCC.

## 1 Introduction

Oral squamous cell carcinoma (OSCC) is the most common type of malignant tumour found in the oral cavity. More than 481,000 new patients are diagnosed annually worldwide [1]. Despite advancements in surgical techniques, adjuvant radiotherapy, and chemotherapy, the overall 5-year survival rate of OSCC patients is approximately 50%–60% [[1], [2], [3]]. Cigarette smoking, alcohol drinking, betel chewing, and gene mutation are major risk factors for OSCC [[4], [5], [6]]. However, the occurrence of OSCC in some patients cannot be explained by these risk factors, suggesting the existence of other risk factors [7,8].

The oral cavity, which contains more than one thousand different kinds of microbes, is an important ecological area for microbial colonization and survival in humans [9]. This oral bacterial flora plays an essential role in maintaining a normal oral physiological environment [10,11]. Microbial dysbiosis leads to various diseases, such as dental caries, periodontal disease, and oral cancer, and seriously affects human life and health [12,13]. In recent years, with the advent of 16S rRNA high-throughput sequencing, there has been increasing interest in the potential role of the microbiome in the occurrence of OSCC [14,15]. This sequencing provides a more comprehensive and accurate method to describe the microbiome during health and disease. To date, studies have found that a number of bacteria in the oral microbiome are associated with OSCC [16]. However, the results of these studies fluctuate owing to differences in design, sample size, and sites. After comparing the mouthwash samples of OSCC patients and healthy controls, a recent study found that the abundances of *Fusobacterium periodonticum* and *Streptococcus constellatus* were positively correlated with the development of OSCC [17]. Another report revealed that *Fusobacterium*, *Alloprevotella* and *Porphyromonas* abundances were enriched after comparing the compositions of the microbiome between OSCC tumour sites and opposite normal tissues [18]. However, another study found that *Streptococcus gorgoniid*, a health-associated species, antagonizes the pro-cancer phenotypes induced by *Porphyromonas gingivalis*, highlighting the need to explore the protective function of the relationship between the microbiome and oral cancer [19].

Most of these studies have explored the dysbiosis via comparing oral microbiome between the patients with OSCC and healthy individuals or between the cancerous tissues and paired paracancerous tissues from the same OSCC patients [[20], [21], [23]]. However, there is no consensus among reports on the cancer-associated changes in the oral microbiome. In this systematic review and meta-analysis, we summarized the published scientific literatures and assessed the differences in the oral microbiome between patients with OSCC and healthy controls, and between cancerous tissue and paired paracancerous tissue.

## 2 Materials and methods

### 2.1 Search strategy

This systematic review and meta-analysis were carried out according to Preferred Reporting Items for Systematic Reviews and Meta-Analyses (PRISMA) guidelines [24]. Full-text original research articles on OSCC and oral bacteria were identified in a search of the PubMed, MEDLINE, EMBASE, Cochrane Library, Google Scholar, and Web of Science databases on December 2021. The following keywords were used: “oral cancer OR oral squamous cell carcinoma OR OSCC AND oral microbiome”; “mouth squamous cell carcinoma OR OSCC AND microbiome”; “oral squamous cell carcinoma OR head and neck squamous cell carcinoma AND oral microbiome”; “OSCC OR oral squamous cell carcinoma AND oral bacteriome OR oral microbiome”; “oral squamous cell carcinoma AND oral microbiome AND next-generation sequencing technology OR NGS OR 16S rRNA”.

The inclusion criteria were as follows: (1) studies that investigated the correlation between OSCC and the oral microbiome; (2) studies in which the method used for oral microbiome sequencing was next-generation sequencing technology; (3) case-control studies or studies of paired cancerous and paracancerous tissues from the same OSCC patients; (4) clinical studies in humans; and (5) studies in which the microbiological content of all the samples was evaluated.

The exclusion criteria were as follows: (1) papers from conferences or congresses, systematic reviews, reviews, case reports and meeting abstracts; (2) papers describing studies using animal experiments; (3) papers describing studies on head and neck cancer or oropharyngeal cancer that did not clearly indicate OSCC in the paper; and (4) papers describing data that could not be extracted; (5) The oral microbiome was not analysed using next-generation sequencing technique on the 16S rRNA gene.

### 2.2 Data extraction

Two researchers (Yu and Wang) independently screened the eligible studies, and articles that did not meet the inclusion criteria were excluded. The following data were collected: articles and year of publication, participant age, sample type, population, region of 16S rRNA sequenced, and bacterial alteration in OSCC. To assess changes in the relative abundance at phylum level, the trend of an increase or decrease in the abundance for each of five dominant phyla was extracted. To analyse changes in the relative abundance at the genus level, the mean value was extracted directly from nine included literature. In studies in which the mean value was not directly available [17,22,25,26], it was then extracted from graphs of the original articles as described in a previous study [27]. To calculate standard deviation (SD), the relative abundance of bacteria genera of each sample and total number of samples were extracted from three studies [4,28,29]. *P* value was extracted for calculation of SD from ten studies [17,18,20,22,23,25,26,[30], [31], [32]].

### 2.3 Methodological assessment

These included studies were methodologically evaluated using the Newcastle-Ottawa scale [33]. The maximum score was 7, and studies with a total score of ≥5 points were deemed to be of high quality (Table S1). Two researchers assessed the scores independently, and any disagreements in this respect were resolved by consensus.

### 2.4 Data analysis

To analyse the differences of the relative abundance of bacteria phyla between groups, the rising or decreasing trend was qualitatively assessed using heatmap analyses in GraphPad Prism 9. To calculate SD for three studies [4,28,29] the median value, minimum and maximum values of relative abundance of bacteria genera were firstly calculated. Subsequently, SD was estimated using formula describe in the previous study [34]. For those ten studies from which *P* value were extracted, the SD was first calculated from *t* statistic converted from *P* value extracted. Subsequently SD value was calculated from the standard error (SE) as previously reported [35].

The standardized mean difference (SMD) measure of effect was used for the continuous variables [36]. SMD >0 indicates that the genus of the microbe in the microbiome in patients with OSCC has a higher relative abundance than that in controls, and SMD <0 indicates that the genus of the microbiome in patients with OSCC has a lower bacterial abundance than that in controls.

A random-effect meta-analysis was undertaken using STATA (v17.0), selecting the inverse-variance method. Heterogeneity was assessed by calculating I^2^ [37], assuming a value of 25% to indicate low heterogeneity, 50% as moderate, and 75% as high. Forest plots were used to visualize the results. Sensitivity analysis was also conducted by removing one study at a time to assess the robustness of the results. Publication bias was evaluated by using funnel plots. Statistical significance was set at *P* < 0.05 for all analyses.

## 3 Results

### 3.1 Characteristics of the included studies

**Fig. 1:**
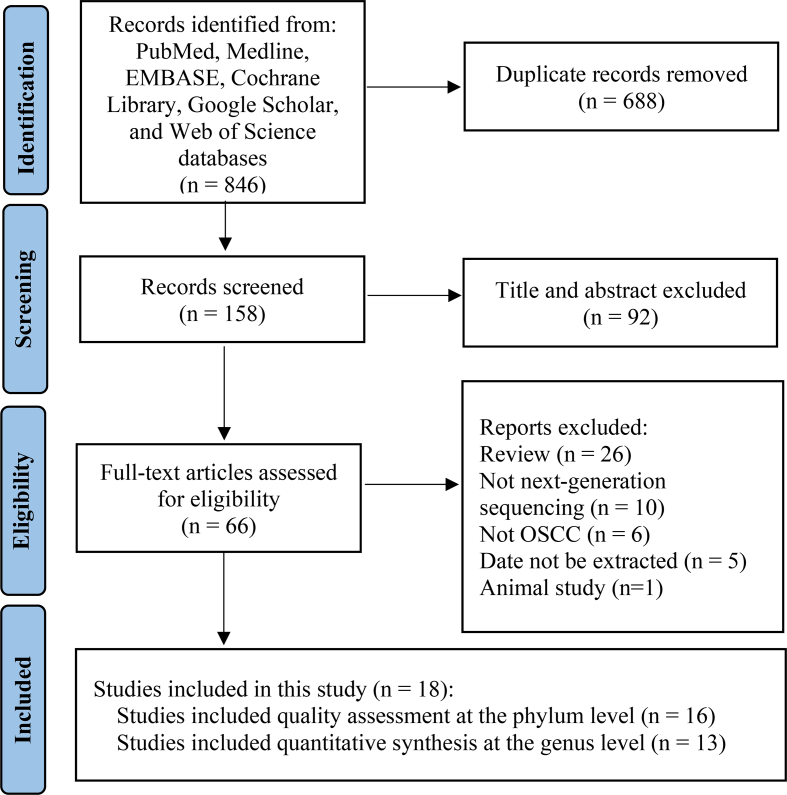
PRISMA flow diagram showing the study selection process.

The selection process of the included studies is shown in Fig. 1. A total of 846 publications were retrieved from the initial literature search, from which 66 full texts were screened for eligibility. Eighteen articles reporting on the oral microbiome in OSCC compared to that in healthy individuals or paired paracancerous tissues as controls were identified. The trend of an increase or decrease from 16 studies was extracted for qualitative assessment of the variations of the relative abundance at the phylum level. Data about the abundance of bacteria genera was extracted from 13 studies and used for meta-analysis.

The characteristics of the included studies, which were conducted between 2014 and 2021, are summarized in Table 1. Sample types included tissues (6 articles), saliva (4 articles), oral swabs (4 articles), and oral rinses (3 articles). In one study, oral swabs from healthy subjects and cancer tissues from OSCC patients were collected. The predominant sequenced regions of 16S rRNA were V3–V4 (8 articles), V4 (5 articles), and V4–V5 (2 articles), with one study each that assessed V1–V3, V3–V5 and V6–V8. The 18 articles included were divided into two categories: 1) case-control studies in which oral microbiome have been compared between patients with OSCC and healthy controls (9 studies); and 2) studies of paired tissues in which oral microbiome in cancerous tissues of OSCC patients have been compared with that in paired paracancerous tissues (9 studies). For the category of case-control studies, there were a total of 443 participants with OSCC and 301 healthy individuals with an age range from 30 to 60 years. For the category of studies of paired tissues, there were 312 patients with OSCC from which paired cancerous tissues and matched paracancerous tissues were collected. Their ages ranged from 50 to 60 years (Table 1). For those 13 articles which were included in the meta-analysis, the total participants in the category of case-control studies were composed of 302 patients with OSCC and 164 healthy individuals, while in the category of studies of paired tissues, the participants were summed to 262 patients.

**Table 1:** Characteristics of the studies included.

Study, Year	Sample Type	Group	Sample Size	Average/Range of Age (Years)	Region of 16S rRNA	Bacterial Alteration in OSCC
Schmidt et al., 2014	Oral Swab	OSCC HC	10 6	51 –	V4	**Phylum:** Actinobacteria↓, Firmicutes↓ **Genus:***Fusobacterium*↑, *Rothia*↓, *Streptococcus*↓
Guerrero-Preston et al., 2016	Saliva	OSCC HC	10 25	–	V3–V5	**Phylum:** Bacteroidetes↑, Fusobacteria↑, Proteobacteria↑ **Genus:***Haemophilus*↑, *Lactobacillus*↑, *Prevotella*↑, *Streptococcus*↑, *Veillonella*↑
Al-Hebshi et al., 2017	Tissues, swab	OSCC HC	20 20	53.6 ± 10.4 52.3 ± 8.9	V1–V3	**Phylum:** Fusobacteria↑, Proteobacteria↑, Actinobacteria↓, Firmicutes↓ **Genus:***Haemophillus*↓, *Rothia*↓, *Streptococcus*↓
Lee et al., 2017	Saliva	OSCC HC	125 127	53 ± 10 52 ± 14	V4	**Genus:***Alistipes*↑, *Bacteroides*↑, *Blautia*↑, *Clostridium*↑, *Dorea*↑, *Escherichia*↑, *Faecalibacterium*↑, *Megamonas*↑, *Phascolarctobacterium*↑
Yang et al., 2018	Oral rinse	OSCC HC	197 51	32–87 22–56	V3–V4	**Phylum:** Fusobacteria↑, Actinobacteria↓, Bacteroidetes↓ **Genus:***Fusobacterium*↑, *Haemophilus*↓, *Neisseria↓*, *Rothia*↓, *Streptococcus*↓, *Veillonella*↓
Ganly et al., 2019	Oral rinse	OSCC HC	18 12	59.8 ± 10.9 44.4 ± 15.6	V3–V4	**Genus:***Alloprevotella*↑, *Fusobacterium*↑, *Prevotella*↑, *Streptococcus*↓
Hashimoto et al., 2019	Saliva	OSCC HC	6 4	50.7 31	V4	**Phylum:** Bacteroidetes↑, Firmicutes↓ **Genus:***Streptococcus*↓
Sawant et al., 2021	Oral rinse	OSCC HC	10 10	–	V6–V8	**Phylum:** Actinobacteria↓, Bacteroidetes↓, Firmicutes↓ **Genus:***Prevotella*↑, *Streptococcus*↓
Zhou et al., 2021	Saliva	OSCC HC	47 46	–	V4–V5	**Phylum:** Actinobacteria↑, Firmicutes↑, Bacteroidetes↓, Proteobacteria↓ **Genus:***Actinobacteria*↑, *Bacillus*↑, *Fusobacterium*↑, *Moraxella*↑, *Veillonella*↑
Zhao et al., 2017	Oral Swab	CT PT	40 40	–	V4–V5	**Phylum:** Bacteroidetes↑, Fusobacteria↑, Actinobacteria↓, Firmicutes↓ **Genus:***Catonella*↑, *Dialister*↑, *Fusobacterium*↑, *Filifacto*r↑, *Parvimonas*↑, *Peptostreptococcus*↑, *Peptococcus*↑
Li et al., 2020	Tissue	CT PT	10 10	61 ± 9.49	V3–V4	**Phylum:** Bacteroidetes↑, Fusobacteria↑, Actinobacteria↓, Proteobacteria↓ **Genus:***Haemophilus*↓, *Neisseria*↓, *Streptococcus*↓
Zhang et al., 2020	Oral Swab	CT PT	50 50	60.7	V3–V4	**Phylum:** Bacteroidetes↑, Fusobacteria↑, Proteobacteria↑ **Genus:***Alloprevotella*↑, *Fusobacterium*↑, *Porphyromonas*↑, *Rothia*↓, *Streptococcus*↓, *Veillonella*↓
Zhou et al., 2020	Tissue	CT PT	24 24	57.2	V3–V4	**Phylum:** Bacteroidetes↑, Firmicutes↑, Fusobacteria↑ **Genus:***Carnobacterium*↑, *Filifactor* ↑, *Fusobacterium*↑, *Parvimonas*↑, *Tannerella*↑, *Peptostreptococcus*↑, *Streptococcus*↑, *Treponema*↑
Su et al., 2021	Oral Swab	CT PT	74 74	53	V4	**Phylum:** Bacteroidetes↑ and Fusobacteria↑, Firmicutes↓ and Proteobacteria↓ **Genus:***Campylobacter*↑, *Capnocytophaga*↑, *Fusobacterium*↑, *Peptostreptococcus*↑, *Prevotella*↑, *Streptococcus*↓
Sarkar et al., 2021	Tissue	CT PT	50 50	52.68	V3–V4	**Phylum:** Bacteroidetes↑, Fusobacteria↑ **Genus:***Corynebacterium*↑, *Deinococcus*↑, *Noviherbaspirillum*↑, *Prevotella*↑, *Pseudomonas*↑, *Actinomyces*↓, *Anoxybacillus*↓, *Serratia*↓, *Stenotrophomonas*↓, *Sutterella*↓
Torralba et al., 2021	Tissue	CT PT	18 18	–	V4	**Genus:***Fusobacterium*↑, *Parvimonas*↑, *Peptostreptococcus*↑, *Porphyromonas*↑, *Prevotella*↑, *Streptococcus*↓, *Veillonella*↓
Yang et al., 2021	Tissue	CT PT	23 23	61.9 ± 12.3	V3–V4	**Phylum:** Bacteroidetes↑, Fusobacteria↑, Firmicutes↓, Proteobacteria↓ **Genus:***Campylobacter*↑, *Catonella*↑, *Filifactor*↑, *Gemella*↑
Ye et al., 2021	Tissue	CT PT	23 23	63 ± 9.6	V3–V4	**Phylum:** Bacteroidetes↑, Actinobacteria↓ **Genus:***Actinomyces*↓, *Streptococcus*↓, *Neisseria*↓

OSCC: oral squamous cell carcinoma; HC: healthy control; CT: cancerous tissues of OSCC; PT: paracancerous tissues of OSCC; –: Not mentioned.

### 3.2 Compositional changes in the microbiome observed in the case-control studies

#### 3.2.1 Summary of the changes in representative phyla

**Fig. 2:**
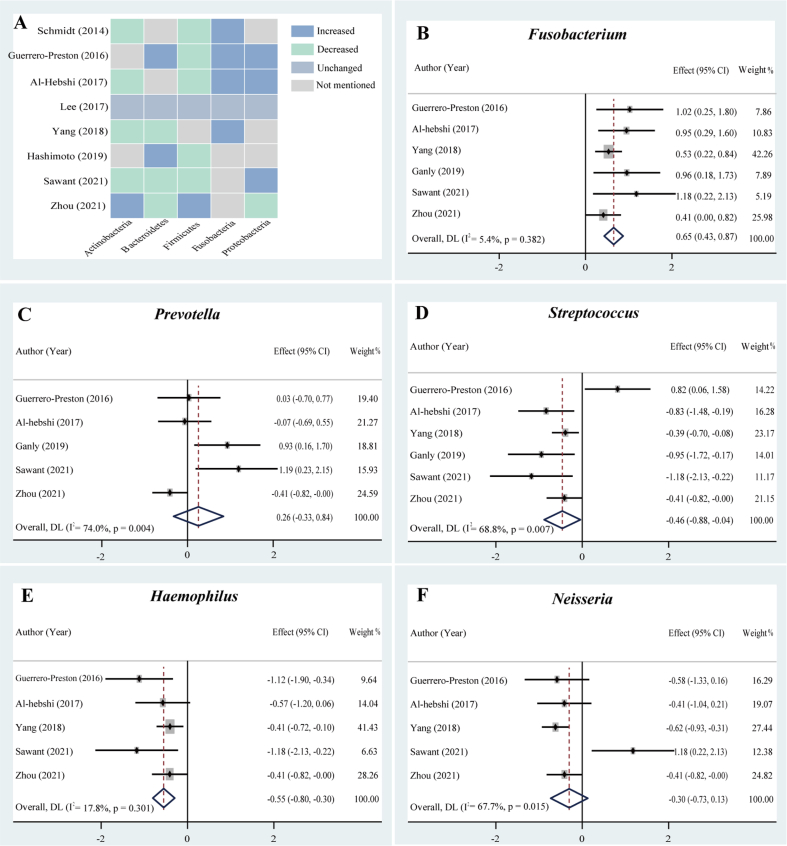
The changes in the composition of the microbiome in patients with OSCC compared with those of healthy controls. (A) Heatmap analyses at the phylum level; forest plots of the relative abundance of bacterial genera using random-effects models, including (B) *Fusobacterium*, (C) *Prevotella*, (D) *Streptococcus*, (E) *Haemophilus*, and (F) *Neisseria*. The squares and horizontal lines represent the value of SMD and 95% CIs, respectively. The size of square boxes is proportional to each study’s weight in the meta-analysis. The diamond represents the combined value of SMD and corresponding 95% CIs. Effect value (combined value of SMD) > 0 indicates the genus has a higher relative abundance in OSCC, and vice versa.

Eight studies were included to analyse the differences in the representative phyla of the microbiome in patients with OSCC and those of healthy individuals. The trends of the relative abundances of Fusobacteria (80%, 4/5) and Proteobacteria (60%, 3/5) were increased in the OSCC group (Fig. 2A). The trends of the relative abundances of Actinobacteria (66.7%, 4/6), Firmicutes (71.4%, 5/7) and Bacteroidetes (50%, 3/6) in the OSCC group were lower than those in the healthy control group. These findings were consistent with the trends of the relative abundance of the five phyla of the OSCC microbiome in most included studies.

#### 3.2.2 Bacterial genera enriched in OSCC

##### 3.2.2.1 Fusobacterium

In the meta-analysis of *Fusobacterium* abundance, six studies were included. The results of the forest plot showed that the relative abundance of *Fusobacterium* was higher in the OSCC group than in the healthy control group (n = 6, SMD = 0.65, 95% CI: 0.43–0.87, I^2^ = 5.4%) (Fig. 2B). The overall effect size was significant and large (Z = 5.809, *P* = 0.000). It was suggested that the enriched *Fusobacterium* abundance was closely associated with OSCC.

##### 3.2.2.2 Prevotella

Five studies were included in the meta-analysis. As shown in Fig. 2, the forest plot indicated that the relative abundance of *Prevotella* in the OSCC group was higher than that in the healthy control group (n = 5, SMD = 0.26, 95% CI: −0.33–0.84, I^2^ = 74.0%), with high heterogeneity (Fig. 2C). However, its overall effect size was relatively small, and the result was not statistically significant (Z = 0.869, *P* = 0.398).

#### 3.2.3 Bacterial genera depleted in OSCC

##### 3.2.3.1 Streptococcus

Six studies were included in the meta-analysis of *Streptococcus* abundance. The forest plot showed that the relative abundance of *Streptococcus* in the OSCC group was lower than that in the healthy group (n = 6, SMD = −0.46, 95% CI: −0.88∼-0.04, I^2^ = 68.8%) with slightly high heterogeneity (Fig. 2D). The overall effect size was significant and moderate (Z = −2.146, *P* = 0.032). Although the heterogeneity of the results was slightly high, the depletion in *Streptococcus* abundance was closely linked to OSCC.

##### 3.2.3.2 Haemophilus

Five studies were included in the meta-analysis of *Haemophilus* abundance. As shown in Fig. 2, the relative abundance of *Haemophilus* in the OSCC group was lower than that in the healthy control group (n = 5, SMD = −0.55, 95% CI, −0.80−0.30, I^2^ = 17.8%), with lower heterogeneity (Fig. 2E). Its overall effect size (Z = −4.243, *P* = 0.000) was relatively large and significant. It was suggested that the depleted *Haemophilus* abundance was closely related to OSCC.

##### 3.2.3.3 Neisseria

Six studies were included in the meta-analysis of *Neisseria* abundance. The forest map results showed that the relative abundance of *Neisseria* was lower in the OSCC group than in the healthy control group (n = 6, SMD = −0.30, 95% CI, −0.73–0.13, I^2^ = 67.7%) with high heterogeneity (Fig. 2F). However, its overall effect size (Z = −1.373, *P* = 0.170) was relatively small, and the results were not statistically significant.

### 3.3 Compositional changes in the microbiome observed in studies of paired tissues

#### 3.3.1 Summary of changes in the representative phyla

**Fig. 3:**
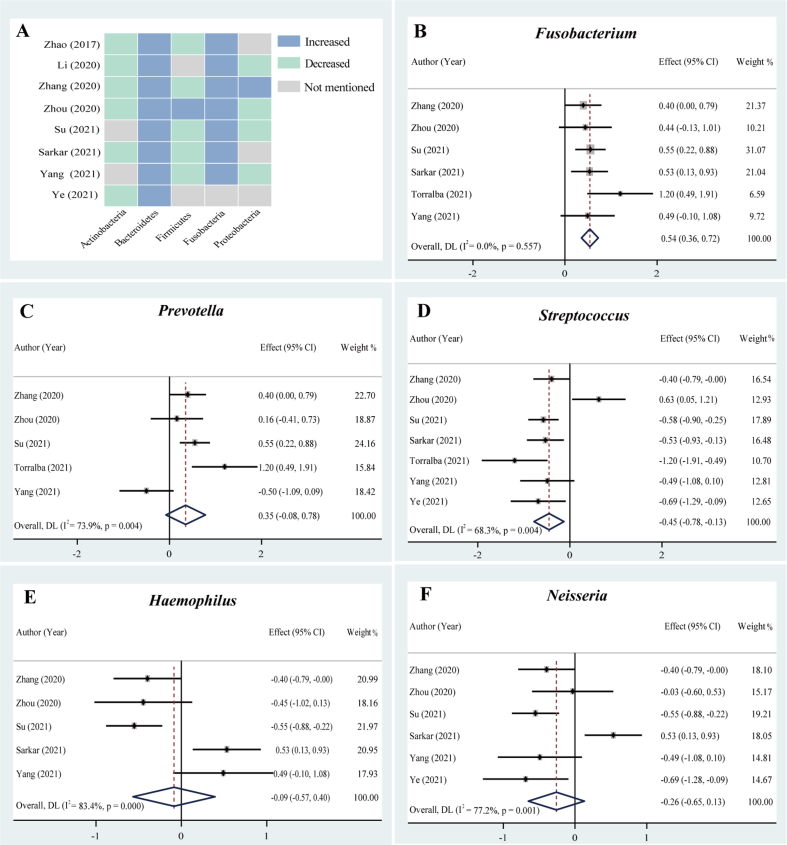
The changes in the composition of the microbiome in cancerous tissues with OSCC compared with those from paired paracancerous tissues. (A) Heatmap analyses at the phylum level; forest plots of the relative abundance of bacterial genera using random-effects models, including (B) *Fusobacterium*, (C) *Prevotella*, (D) *Streptococcus*, (E) *Haemophilus*, and (F) *Neisseria*. The squares and horizontal lines represent the value of SMD and 95% CIs, respectively. The size of square boxes is proportional to each study’s weight in the meta-analysis. The diamond represents the combined value of SMD and corresponding 95% CIs. Effect value (combined value of SMD) > 0 indicates the genus has a higher relative abundance in cancerous tissue, and vice versa.

Eight studies were included. The trends of the relative abundances of Fusobacteria (100%, 7/7) and Bacteroidetes (100%, 8/8) were both increased in the OSCC group (Fig. 3A). The trends of the relative abundances of Actinobacteria (100%, 6/6), Firmicutes (83.3%, 5/6) and Proteobacteria (80%, 4/5) in the OSCC group were lower than those in the paired paracancerous group.

#### 3.3.2 Bacterial genera enriched in OSCC

In the meta-analysis of *Fusobacterium* abundance, we included six studies. The results of the forest plots showed the relative abundance of *Fusobacterium* was higher in the cancerous group than in the paired paracancerous group (n = 6, SMD = 0.54, 95% CI: 0.36–0.72, I^2^ = 0.0%) (Fig. 3B). The overall effect size was significant and large (Z = 5.785, *P* = 0.000). The results indicate that the enriched *Fusobacterium* abundance may be closely related to the occurrence of OSCC.

Five studies were included in the meta-analysis of *Prevotella* abundance, and the relative abundance in the cancerous group was higher than that in the paired paracancerous group. However, the results of the overall effect were not statistically significant (Fig. 3C).

#### 3.3.3 Bacterial genera depleted in OSCC

In the meta-analysis of *Streptococcus* abundance, seven studies were included. The results of the forest plot showed that the relative abundance of *Streptococcus* in the cancerous group was lower than that in the paired paracancerous group (n = 7, SMD = −0.45, 95% CI: −0.78–0.13, I^2^ = 68.3%) with slightly high heterogeneity (Fig. 3D). The overall effect size was significant and moderate (Z = −2.726, *P* = 0.006). It was suggested that *Streptococcus* abundance was depleted in the microbiome of OSCC.

The meta-analysis of *Haemophilus* and *Neisseria* abundances included five and six studies, respectively. The results of the forest plots showed that the relative abundances of both *Haemophilus* and *Neisseria* in the cancerous group were lower than those in the paired paracancerous group (Fig. 3E–F). However, the overall effect sizes were not statistically significant.

### 3.4 Sensitivity analysis and publication bias

The results of the sensitivity analysis demonstrated that the SMD was not significantly influenced by any single study, as indicated by the analysis performed after the sequential omission of one study at a time (Figs. S1 and S2). A funnel plot (Figs. S3 and S4) indicated a lack of significant publication bias.

## 4 Discussion

Dysbiosis of the microbiome is closely associated with OSCC [38]. Previous studies confirmed alterations in the oral microbiome of OSCC, but the results were controversial [16]. Quantitatively analysing the compositional alterations in the oral microbiome of OSCC using a meta-analysis have not been performed yet to resolve the issue. In this study, we conducted a systematic review and used a meta-analysis to pool the results of 18 studies with fair and high-quality ratings and to provide joint information on the relative abundances of bacteria at the phylum and genus levels in 755 patients with OSCC and 613 controls.

At the phylum level, the trends of the enrichment for Fusobacteria and decreased Actinobacteria and Firmicutes abundances in the OSCC microbiome were consistent with those observed in healthy control individuals or paired paracancerous tissues, while different trends were observed for Bacteroidetes and Proteobacteria abundances. The contrasting trends might reflect the content variations in these phyla. It has been shown Bacteroidetes Cluster 1 is decreased in colorectal cancer (CRC), whereas the combined abundance of Bacteroidetes Cluster 2 and Prevotella Cluster is increased [39]. Variation of the relative abundance of Proteobacteria has been found in cancerous tissues of CRC [40]. Owing to the fact that this was a qualitative analysis at the phylum level, further research still needs to be performed to acquire enough data on relative abundances from published papers.

At the genus level, the results of the meta-analysis demonstrated that the abundances of *Fusobacterium* and *Prevotella* were higher, while the abundances of *Streptococcus*, *Haemophilus* and *Neisseria* were lower in the OSCC microbiome than in healthy control individuals. Among them, the changes in the abundances of *Fusobacterium*, *Streptococcus* and *Haemophilus* were statistically significant in the OSCC microbiome. The above findings were further confirmed in comparisons made between OSCC tissues and paired paracancerous tissue samples, except for the findings regarding the abundance of *Haemophilus*. Additionally, we found that the alterations in the genera of the microbiome of OSCC in the above two types of studies were very similar. These findings were also supported by a study comparing the microbiome in gastric tumour tissues with those of paracancerous samples or superficial gastritis samples [41]. Our results demonstrated that enriched *Fusobacterium* and depleted *Streptococcus* abundances may participate in the occurrence and development of OSCC.

*Fusobacterium*, *Streptococcus*, *Prevotella*, *Haemophilus* and *Neisseria* are closely related to periodontitis and OSCC [42]. Even though the mechanisms underlying bacterial involvement in OSCC remain unknown, it has been reported that the process of oral bacterial infection is closely related to periodontitis, which eventually leads to OSCC [43]. Two meta-analyses reported an approximately 2- to 5-folds higher risk for OSCC in patients with periodontitis than that in those without periodontitis [44]. Various periodontal bacteria, such as *Fusobacterium nucleatum* and *Prevotella intermedia*, are related to OSCC [44,45].

Increased abundance of *Fusobacterium* plays has also been found in cancerous tissues in CRC [40,46]. This genetically diverse bacterium possesses a number of virulence factors including the type V secretion system, adhesins and lipopolysaccharide, which are involved in the carcinogenesis of colon [40,46]. Meanwhile, *Fusobacterium* spp. was also shown to promote the development of OSCC in vitro by regulating the mRNA and protein expression levels of interleukin (IL)-6 and IL-8, matrix metallopeptidase 9 (MMP-9) and Cyclin-D1 [47,48]. Inflammatory cytokines, such as IL-6, IL-8, tumour necrosis factor alpha (TNF-ɑ) and MMP, are thought to promote carcinogenesis by inducing mutations, genomic instability, and epigenetic changes. Signal transducer and activator of transcription 3 (STAT3), nuclear factor kappa B (NF-κB) and other key factors in precancerous cells promote malignant processes, including proliferation, angiogenesis, invasion, and metastasis [47,49].

*F. nucleatum*, which is one of the most representative species of *Fusobacterium*, its genomes harbour bacterial virulence and adhesion factors, such as FadA (*Fusobacterium* adhesin A), Fap2 (*Fusobacterium* autotransporter protein 2), and CbpF (CEACAM binding protein of *Fusobacterium*) [46,50]. FadA has been found to bind to E-cadherin on colon cancer cells and in turn activate the WNT/β-catenin signalling pathway. The mRNA expression levels of FadA in the OSCC-bacteria-positive patients were significantly higher than those in the control group [51]. It was suggested that FadA of *F. nucleatum* may also serve as a new target for therapeutic intervention of oral cancer. Fap2 selectively attaches to Gal-GalNAc (Galβ1-3GalNAc) on cancer cells and markedly enhances *F. nucleatum* presence and abundance in the tumours. A recent study revealed that Fap2 also bind to T cell immunoreceptors with Ig and immunoreceptor tyrosine-based inhibitory motif domains (TIGIT), and impair T and NK cell cytotoxicity, and promote immune cell death, resulting in tumour escape from immunosurveillance [52,53]. Furthermore, Fap2, as a galactose-sensitive adhesion factor, can mediate co-adherence of neighbouring bacteria and increase the diversity and the stability of the developing dental plaque in the oral cavity [54]. However, the role of Fap2 to the development of OSCC requires further research. Recent evidence suggests that *F. nucleatum* uses its trimeric autotransporter adhesin CbpF to inhibit T-cell function by activating CEACAM1 [55]. In addition, CEACAM1 overexpression is associated with OSCC grade and inversely correlated with both overall and disease-specific 5-year survival [56]. Therefore, *F. nucleatum* may potentially drive the progression of oral cancer via multifunctional adhesion and needs to be explored further.

Previous studies have found that both *Streptococcus* and *Haemophilus* are dominant bacteria of the core oral microbiome in healthy individuals [17,57]. Several studies have reported that *Streptococci*, such as *Streptococcus salivarius* and *Streptococcus cristatus*, inhibit the inflammatory response. *S. salivarius* strain K12 contributes to the host defence process by downregulating inflammatory responses by inhibiting the NF-κB pathway and interfering with IL-8 synthesis and secretion [58]. *S. cristatus* was shown to be able to inhibit *F. nucleatum*-induced proinflammatory responses in oral epithelial cells and appeared to be involved in the blockade of NF-κB nuclear translocation at the level of IκB-α degradation [59]. This finding also supported our results of enriched *Fusobacterium* and depleted *Streptococcus* abundances in OSCC.

A recent study reported that *Fusobacterium*, *Peptostreptococcus*, and *Prevotella* were abundant in cancerous tissues of gingival squamous cell carcinoma, while *Streptococcus*, *Neisseria*, and *Haemophilus* were abundant in normal buccal mucosa [60]. *Haemophilus arainfluenzae* showed significantly less cytotoxicity against noncancerous telomerase immortalized gingival keratinocyte (TIGK) cells than against OSCC cells. Whether this process may play a protective role against oral cancer needs further investigation [61,62].

In addition, compositional variations in the oral microbiome are related to the mutational changes in OSCC. A significantly higher prevalence of Firmicutes has been found in mutation cluster MSC2, which is characterized by frequent Wnt mutations. Enrichment of Actinobacteria has been found in MSC1, which is characterized by frequent TP53 mutations [17]. The oral microbiome and its products can modulate signalling pathways and gene regulation, and then act as triggers or regulators of genetic and epigenetic changes in the process of tumorigenesis of OSCC [63].

Several limitations of this systematic review should be considered. First, there was a small number of studies included, owing to the difficulty of data extraction. We could not conduct a meta-analysis of alpha diversity (such as Shannon index, Chao1 index, Simpson index) or beta diversity due to the small amounts of eligible studies and insufficient extracted data. We only analysed the relative abundances of bacteria at the genus level due to the insufficiency of data for various bacterial taxonomies. Second, the age and sex of the included subjects, the lifestyle of the subjects and the tumour sites and size of OSCC might contribute to increasing the heterogeneity of the present study. Specially, the microbiome may be influenced by different tumour sites of OSCC, yet some of the included studies did not mention this. Different regions of 16S rRNA have been used for sequencing in the included studies, which might lead to bias at some extent in the taxon classification. This should be taken into account in the future studies to achieve better consistency in the findings between studies. Third, only one study mentioned the oral microbiome of different OSCC stages. Future studies should investigate several factors that could affect the oral bacterial composition, including lifestyle, sites, and stages of OSCC, and sequencing region.

## 5 Conclusions

Our systematic review and meta-analysis summarized the microbial characteristics of OSCC. The alteration of the microbiome profile in patients with OSCC exhibited enriched *Fusobacterium* and decreased *Streptococcus* abundances, which were distinct from those observed in healthy individuals, with these features being evident in both cancerous and paracancerous tissues. Disturbances in the interaction between *Fusobacterium* and *Streptococcus* may prompt the occurrence and development of OSCC.

## Declarations

### Author contribution statement

All authors listed have significantly contributed to the development and the writing of this article.

### Funding statement

This work was supported by Grant from Qingdao Medical Talents Training Program [VYQ2020Y02].

### Data availability statement

Data included in article/supplementary material/referenced in article.

### Declaration of interest’s statement

The authors declare no conflict of interest.
