# Supplementary material for: Microbial dysbiosis in oral squamous cell carcinoma: A systematic review and meta-analysis
Source: Heliyon. 2023 Jan 24;9(2):e13198. doi: 10.1016/j.heliyon.2023.e13198 (PMC9922960; doi:10.1016/j.heliyon.2023.e13198)

**Table S1** Newcastle-Ottawa scale showing quality evaluation of studies included.

| **Publications** | **Selection** | | | |  | **Comparability** |  | **Exposure** | | | **Total** |
| --- | --- | --- | --- | --- | --- | --- | --- | --- | --- | --- | --- |
|  | Definition of cases | Representativeness of the cases | Selection of Controls | Definition of Controls |  | Comparability of cases and controls |  | Ascertainment of exposure | Same method of ascertainment for cases and controls | Non-Response rate |  |
| Schmidt et al., 2014[6] | 1 | 1 | 0 | 1 |  | 1 |  | 1 | 1 | 0 | 6 |
| Guerrero-Preston et al., 2016[28] | 1 | 1 | 0 | 1 |  | 2 |  | 1 | 1 | 0 | 7 |
| Al-Hebshiet al., 2017 [4] | 1 | 1 | 0 | 1 |  | 2 |  | 1 | 1 | 0 | 7 |
| Lee et al., 2017 [5] | 1 | 1 | 0 | 1 |  | 2 |  | 1 | 1 | 0 | 7 |
| Yang et al., 2018 [17] | 1 | 1 | 0 | 1 |  | 1 |  | 1 | 1 | 0 | 6 |
| Ganly et al., 2019 [20] | 1 | 1 | 0 | 1 |  | 2 |  | 1 | 1 | 0 | 7 |
| Hashimoto et al., 2019 [8] | 1 | 1 | 0 | 1 |  | 2 |  | 1 | 1 | 0 | 7 |
| Sawant et al., 2021 [26] | 1 | 1 | 0 | 1 |  | 2 |  | 1 | 1 | 0 | 7 |
| Zhou et al., 2021 [23] | 1 | 1 | 0 | 1 |  | 1 |  | 1 | 1 | 0 | 6 |
| Zhao et al., 2017 [3] | 1 | 1 | 0 | 1 |  | 1 |  | 1 | 1 | 0 | 6 |
| Li et al., 2020 [10] | 1 | 1 | 0 | 1 |  | 2 |  | 1 | 1 | 0 | 7 |
| Zhang et al., 2020 [18] | 1 | 1 | 0 | 1 |  | 1 |  | 1 | 1 | 0 | 6 |
| Zhou et al., 2020 [29] | 1 | 1 | 0 | 1 |  | 1 |  | 1 | 1 | 0 | 6 |
| Su et al., 2021 [25] | 1 | 1 | 0 | 1 |  | 1 |  | 1 | 1 | 0 | 6 |
| Sarkar et al., 2021 [30] | 1 | 1 | 0 | 1 |  | 1 |  | 1 | 1 | 0 | 6 |
| Torralba et al., 2020 [22] | 1 | 1 | 0 | 1 |  | 2 |  | 1 | 1 | 0 | 7 |
| Yang et al., 2021 [31] | 1 | 1 | 0 | 1 |  | 2 |  | 1 | 1 | 0 | 7 |
| Ye et al., 2021 [32] | 1 | 1 | 0 | 1 |  | 1 |  | 1 | 1 | 0 | 6 |

**Figure S1** Sensitivity analyses of bacterial genera of the microbiome in patients with OSCC compared with those of healthy controls, including (A) *Fusobacterium*, (B) *Prevotella*, (C) *Streptococcus*, (D) *Haemophilus*, (E) *Neisseria*.

**Figure S2** Sensitivity analyses of bacterial genera of the microbiome in cancerous tissues with OSCC compared with those from paired paracancerous tissues, including (A) *Fusobacterium*, (B) *Prevotella*, (C) *Streptococcus*, (D) *Haemophilus*, (E) *Neisseria*.

**Figure S3** Funnel plots of publication bias for bacterial abundance of the microbiome in patients with OSCC compared with those of healthy controls, including (A) *Fusobacterium*, (B) *Prevotella*, (C) *Streptococcus*, (D) *Haemophilus*, (E) *Neisseria*.

**Figure S4** Funnel plots of publication bias for bacterial abundance of the microbiome in cancerous tissues with OSCC compared with those from paired paracancerous tissues, including (A) *Fusobacterium*, (B) *Prevotella*, (C) *Streptococcus*, (D) *Haemophilus*, (E) *Neisseria*.

**Figure S1**


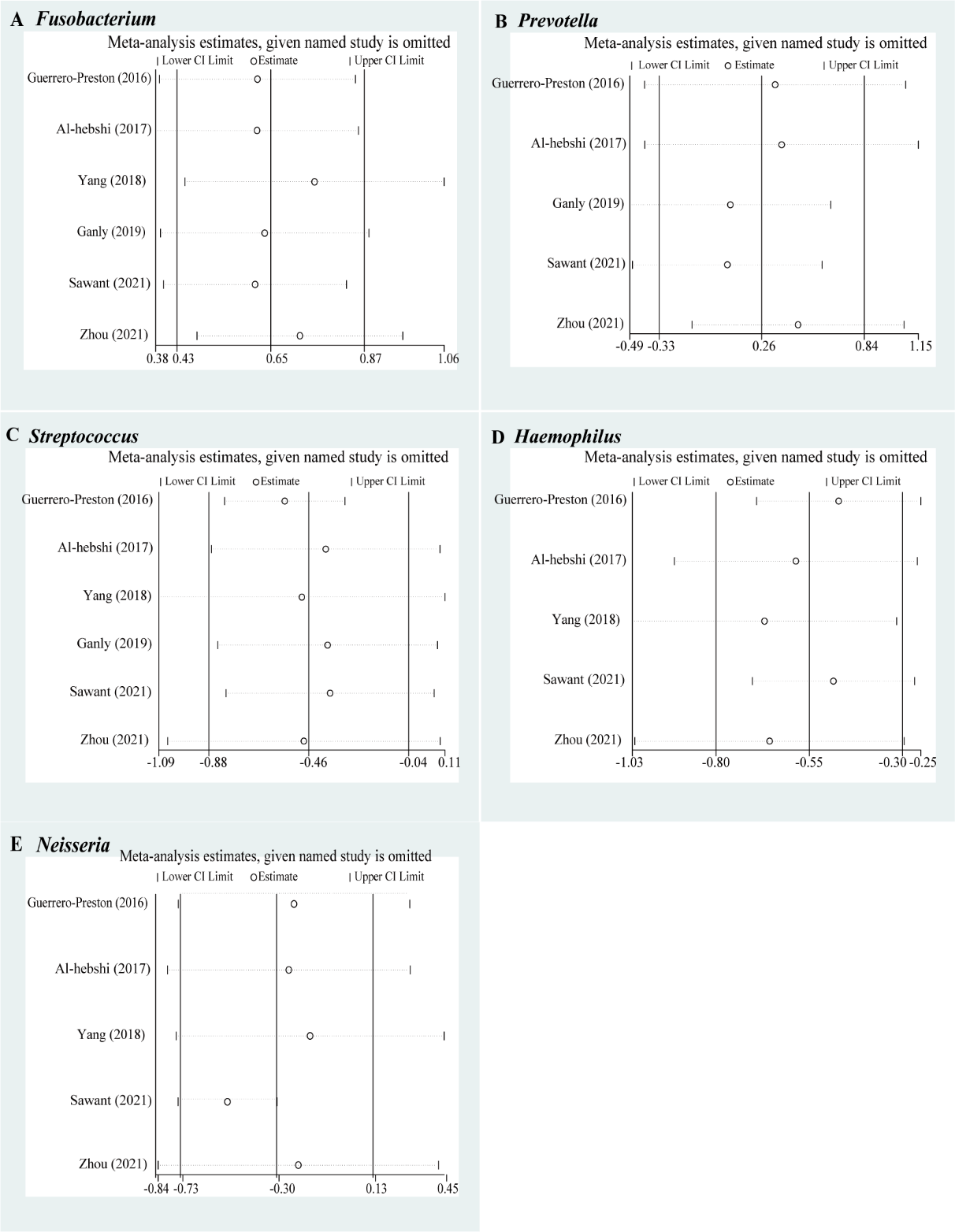


**Figure S2**


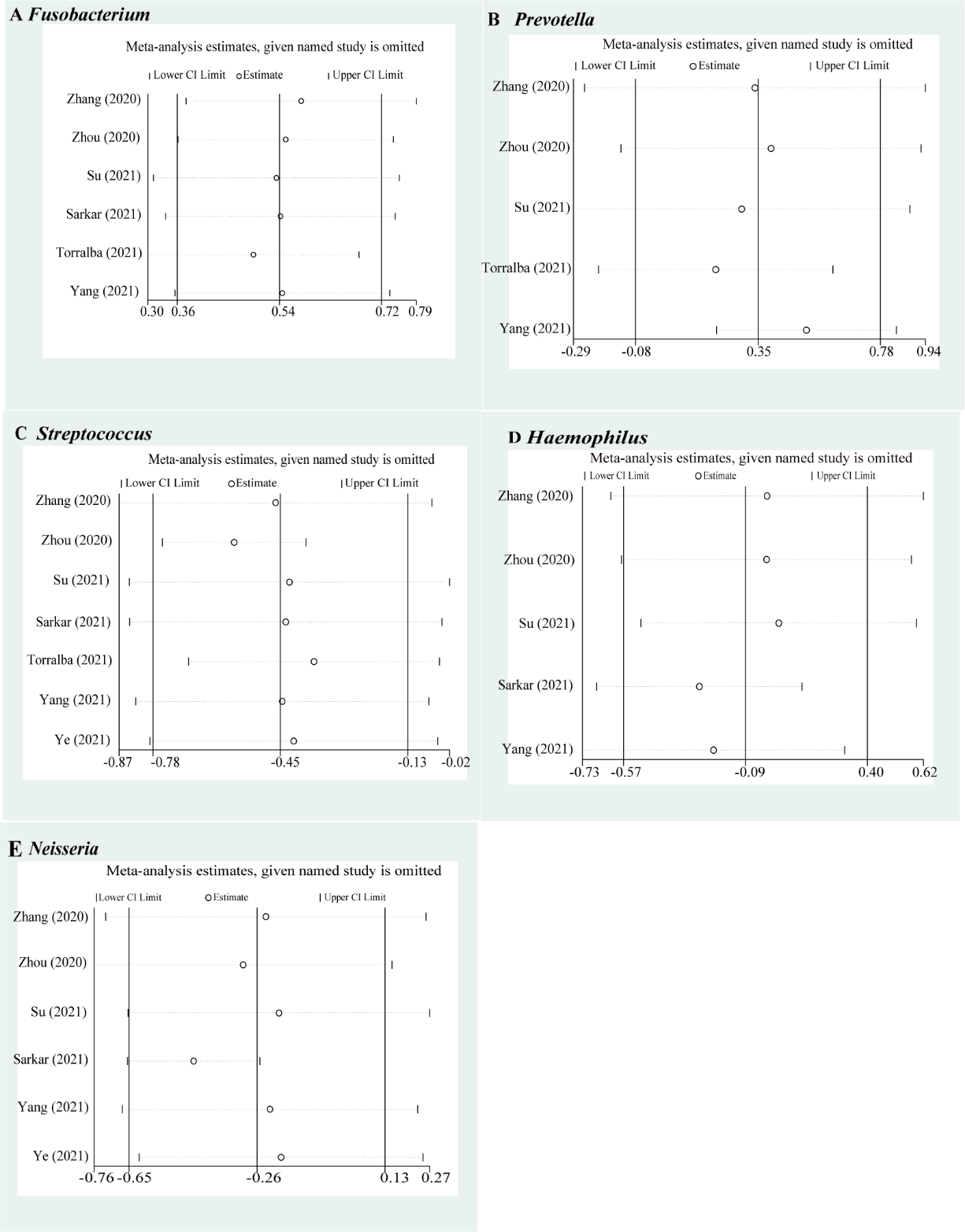


**Figure S3**


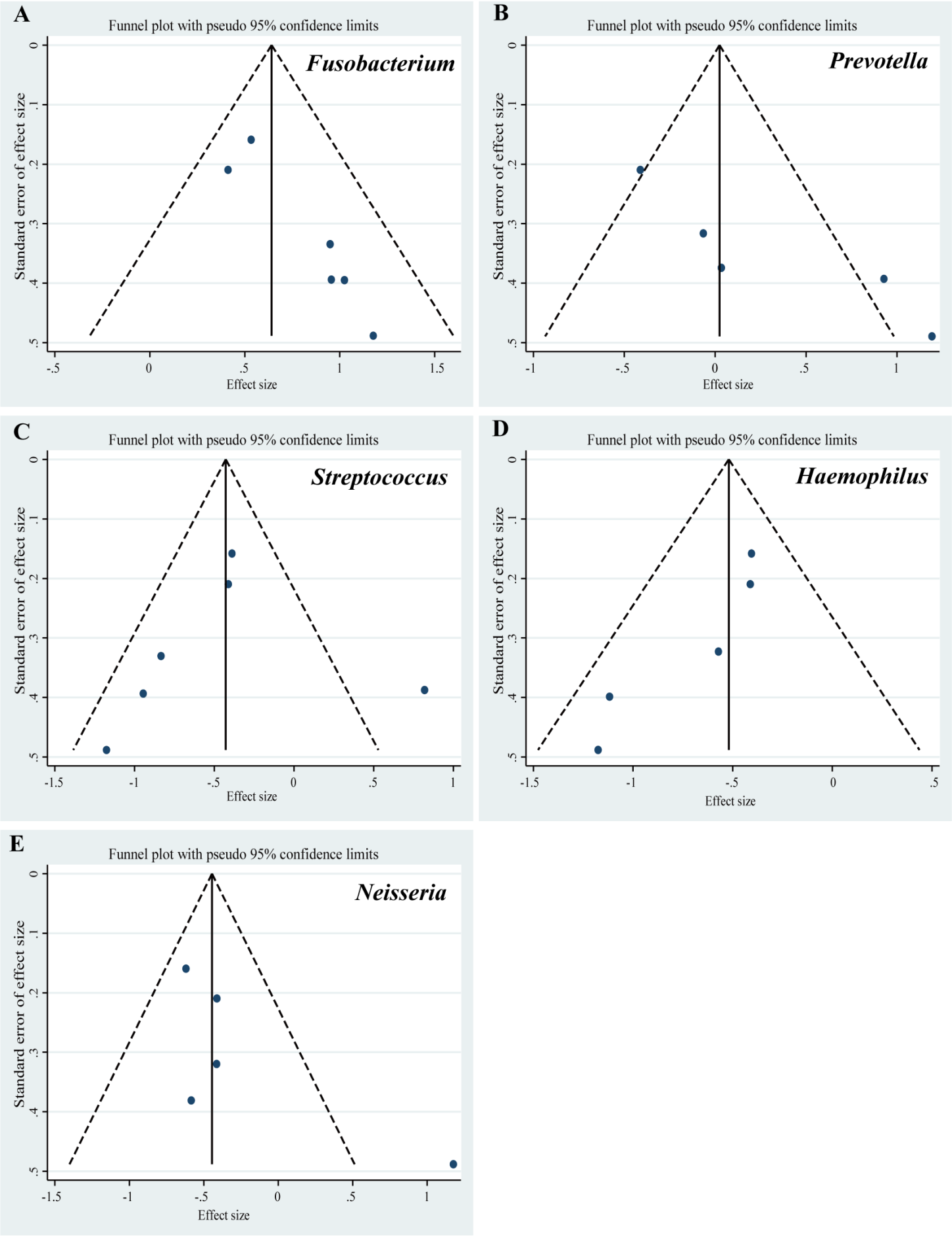


**Figure S4**


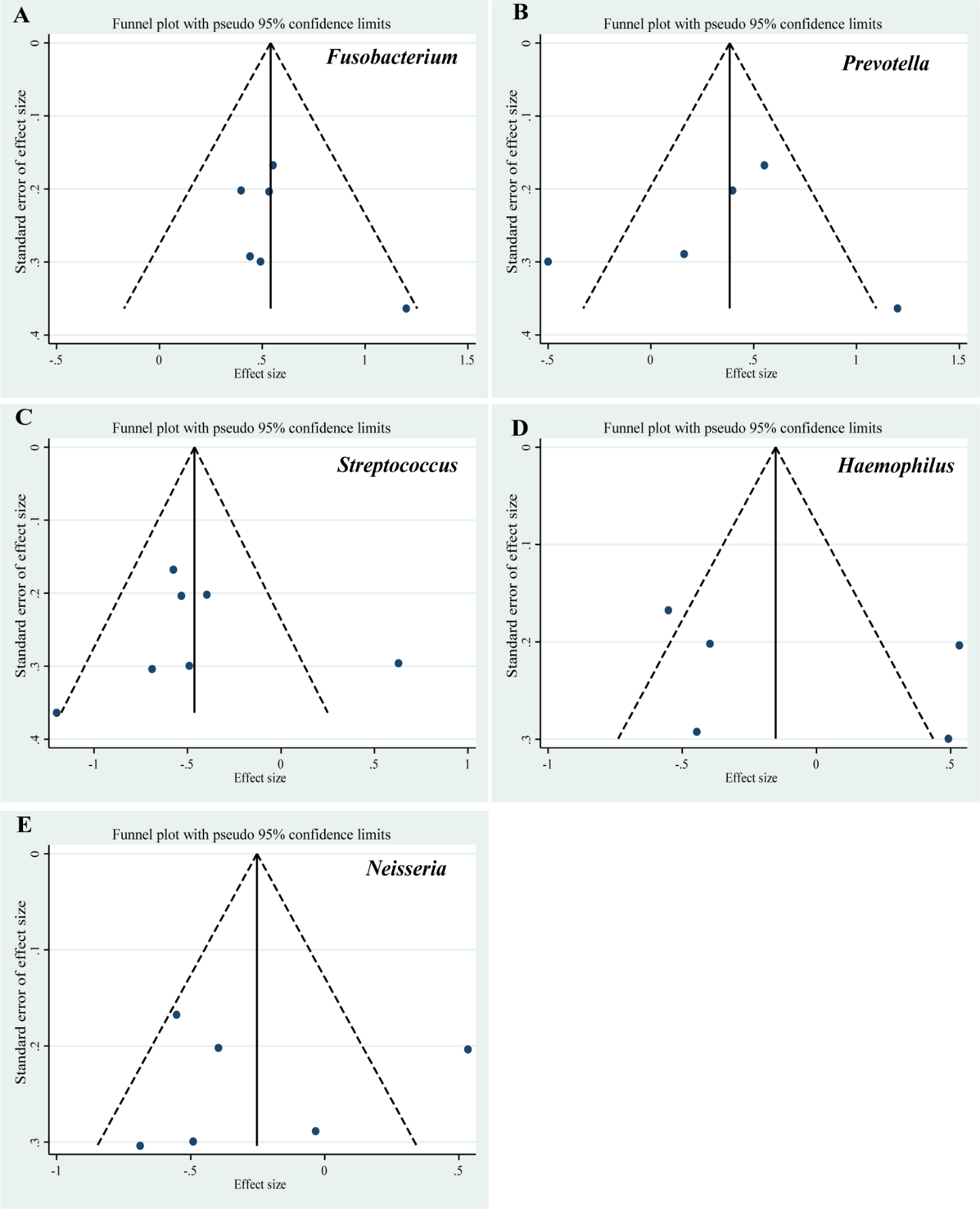

Supplement: Multimedia component 1 [file mmc1.docx]
